# The prevalence of psychosocial related terminology in chiropractic program courses, chiropractic accreditation standards, and chiropractic examining board testing content in the United States

**DOI:** 10.1186/s12998-020-00332-7

**Published:** 2020-08-21

**Authors:** Jordan A. Gliedt, Patrick J. Battaglia, Benjamin D. Holmes

**Affiliations:** 1 Medical College of Wisconsin, Department of Neurosurgery, Milwaukee, WI USA; 2 Logan University Health Centers – Integrative Clinics, Chesterfield, MO USA; 3 Mayo Clinic, Rochester, MN USA

**Keywords:** Chiropractic education, Chiropractic curriculum, Biopsychosocial, Social determinants of health

## Abstract

**Background:**

Spine related disorders entail biological (somatic), psychological, and social factors. Though biological factors are often emphasized, psychosocial considerations may not be receiving proper attention in the chiropractic field. Chiropractors treat spine complaints and therefore should be trained in the full spectrum of the biopsychosocial model. This study examines the use of psychosocial related terminology in United States doctor of chiropractic program (DCP) curricula, the Council on Chiropractic Education (CCE) standards, and the National Board of Chiropractic Examiners (NBCE) test plans.

**Methods:**

Nineteen academic course catalogs, CCE curricular standards and meta-competencies, and NBCE test plans were studied. Terms containing “psycho”, “soci”, “mental”, “econom”, “cultur”, “emotion”, “determinant”, “public”, “communit”, “neighbor”, “behav”, or “cognitive” were identified in each document. Frequency of use, context of use, thematic categorization, and percentage of use compared to overall content were calculated and described.

**Results:**

‘Public’ is the most commonly used psychosocial related term in DCP curricula. ‘Determinant’ was used in 1 DCP curriculum. The number of courses with psychosocial related terminology in course titles and course descriptions ranged from 1 to 5 and 3 to 12, respectively. Most terms are found in clinical skills, special populations, and other miscellaneous courses, with fewer terms found in psychology and public health courses. Terminology use in course titles and descriptions compared to overall content ranges from 3.40 to 14.86%. CCE uses terminology 17 times across 5 (out of 8) total meta-competencies. NBCE includes terminology in test plans I and II, but not III or IV.

**Conclusions:**

Despite evidence suggesting the influential role of psychosocial factors in determinants of health and healthcare delivery, these factors are poorly reflected in United States DCP curricula. This underappreciation is further evidenced by the lack of representation of psychosocial terminology in NBCE parts III and IV test plans. The reasons for this are theoretical; lack of clarity or enforcement of CCE meta-competencies may contribute.

## Background

Chiropractors are recognized by the public as clinicians who care for spine related disorders (SRDs) [1, 2]. SRDs are characterized by a complex interplay between biological (somatic), psychological, and social factors [3, 4], often leading to pain and disability. The biopsychosocial (BPS) model explains this multidimensional nature of SRDs. Biological factors are often emphasized in the clinical evaluation and management of SRDs. However, current best practices incorporate the BPS model, requiring that clinicians identify and address broader modifiers of patients’ health, conventionally termed “psychosocial factors” [5]. Despite these guidelines, adoption of the BPS model in chiropractic care settings is arguably incomplete [6, 7]. Furthermore, students across various healthcare disciplines, including chiropractic, have a poor awareness of these psychosocial factors [8].

Given the above, it is important to understand the extent to which psychosocial determinants of health are taught within doctor of chiropractic program (DCP) curricula. DCPs in the United States are accredited by the Council on Chiropractic Education (CCE), whose purpose is to “promote academic excellence and to ensure the quality of chiropractic education” [9]. One of the primary mechanisms for ensuring educational quality is establishing various meta-competencies for graduates of a CCE-accredited DCP. CCE meta-competencies are described as a set of broad skills and competencies that students must exhibit to satisfy evidence that the DCPs curricular objectives have been met.

Additionally, in the United States, DCP graduates must pass a standardized four-part examination to be eligible for state licensure. These examinations are administered by the National Board of Chiropractic Examiners (NBCE). NBCE parts I and II cover basic and clinical sciences. Parts III and IV involve testing on clinical competencies and case management. Testing categories (termed “test plans”) are available online at https://mynbce.org/. It is reasonable to conclude that United States DCP curricula are modeled to meet CCE meta-competencies for accreditation purposes and reflect NBCE testing content.

The purpose of this study is to examine United States DCP curricula, CCE meta-competencies, and NBCE test plans to determine the use of psychosocial related terminology. This information may identify opportunities for improvement in curricular content, aligning course content with the current best evidence, and preparing chiropractic students to engage in high quality patient care.

## Methods

This study was modeled after previously completed studies investigating prevalence of the term ‘subluxation’ in DCP curricula [10, 11]. Academic course catalogs of DCPs in the United States, CCE accreditation standards for DCPs, and the NBCE testing plans, which are all publicly available, were included in this study. Eligibility criteria for review of DCP course catalogs in this study included English-speaking DCPs with a physical campus location in the United States. The Association of Chiropractic Colleges list of schools was utilized to assist in identifying eligible DCPs [12]. We also identified and included Sherman College of Chiropractic in this study, which is not listed on the Association of Chiropractic Colleges list of schools. We included Sherman College of Chiropractic because, analogous to the DCPs listed on the Association of Chiropractic Colleges list of schools, graduating students are eligible to sit for NBCE testing and thus obtain state licensure and practice as a chiropractor in the United States. A list of all identified institutions with a DCP included in this study is shown in Table 1.

**Table 1:** Institutions with a DCP in the United States

Institution	Location(s)
Cleveland University Kansas City	Overland Park, Kansas, United States
D’Youville College	Buffalo, New York, United States
Keiser University	West Palm Beach, Florida, United States
Life Chiropractic College West	Hayward, California, United States
Life University	Marietta, Georgia, United States
Logan University	Chesterfield, Missouri, United States
National University of Health Sciences	Lombard, Illinois, United States
	Seminole, Florida, United States
New York Chiropractic College	Seneca Falls, New York, United States
Northwestern Health Sciences University	Bloomington, Minnesota, United States
Palmer College of Chiropractic	Davenport, Iowa, United States
	Port Orange, Florida, United States
	San Jose, California, United States
Parker University	Dallas, Texas, United States
Sherman College of Chiropractic	Spartanburg, South Carolina, United States
Southern California University of Health Sciences	Whittier, California, United States
Texas Chiropractic College	Pasadena, Texas, United States
Western States University	Portland, Oregon, United States
University of Bridgeport	Bridgeport, Connecticut, United States

Each institution’s website was searched for an academic course catalog. Catalogs for each analyzed DCP were no older than the 2018 academic year. Each institution’s website provided an accessible DCP course catalog in online format and/or Adobe Acrobat portable document format (PDF) [13–32]. Each catalog provided a list of the DCP curriculum, including course titles and descriptions. National University of Health Sciences has two locations with DCPs and provided one curriculum for both locations. Palmer College of Chiropractic has three locations with DCPs with a separate curriculum for each location listed as part of a single academic catalog.

Data were collected between December 2019 and April 2020. Data extraction was independently performed by two authors (JAG, PJB) and entered into a Microsoft Excel spreadsheet and a Microsoft Word table. Any discrepancy or disagreements between authors was resolved by review of a third author (BDH) and group consensus. The “find” function was used to identify psychosocial related terminology included within DCP curricula. Through a consensus process the authors ascertained the scope of psychosocial related terminology for purposes of this study. Based on the a priori agreed upon scope, each DCP’s list of course titles and course descriptions was assessed for psychosocial related terminology containing “psycho”, “soci”, “mental”, “econom”, “cultur”, “emotion”, “determinant”, “public”, “communit”, “neighbor”, “behav”, or “cognitive”. Words and phrases within DCP curricula, identified during our search, but determined not to be psychosocial related were excluded from analysis and are explained in Table 2.

**Table 2:** Terminology identified in DCP curricula not considered psychosocial in nature

Terminology	Context
Associate	
Associated	
Association	
Behavior(s)	If referenced to muscular tissue (e.g. “behavior of smooth muscle”) If referenced to ethical or professional behavior and codes of conduct
Community	If referenced to community-based clinic learning environment If referenced to ethical and marketing practices in the community
Cognitive	If referenced to the mental/recall function of the student If referenced to physical/tissue related explanations of cognition
Culture	If referenced to microbial matter If referenced to culture of the business of health care
Developmental	
Fundamental(s)	
Economic(s)	If referenced to entrepreneurship on behalf of the chiropractor
Governmental	
Intersegmental	
Phi Chi Omega Honor Society	
Psychomotor	
Public	If referenced to the setting of public communications/practice integration/practice management If referenced to the setting of public protection from harmful effects of radiation If referenced to a physical public setting locale
Publication(s)	
Segmental	
Social	If referenced to social media If referenced to the setting of legality and jurisprudence
Sociological	If referenced to historical events related to the chiropractic profession
Supplemental	

Psychosocial related terminology was aggregated and frequency of use was calculated. Psychosocial related terminology was subsequently categorized by frequency of use in course titles and course descriptions. Courses are described as an academic calendar term-long discrete package of learning on a particular topic, such as anatomy. Course titles refer to the topic devoted to the individual course and course descriptions refer to the narrative explanation of the specific learning content related to that course. To prevent double counting, if psychosocial related terms were used more than once in a course title or course description, it was counted only once toward the “number of course titles terminology used in” or “number of course descriptions terminology used in”. If psychosocial related terms were identified in a course title and also identified in the specific course’s corresponding description, it was counted once in the “number of course titles terminology used in” and once in the “number of course descriptions terminology used in” categories.

Identified terminology was organized into thematic groups, and total number of courses with psychosocial related terms used within each thematic group were calculated. Courses were categorized into one of three thematic groups, based on described content: 1) psychology courses, 2) public health courses, and 3) clinical skills/special populations/other courses.

The percentage of courses that included psychosocial related terms within each DCP’s curriculum was calculated by dividing the number of individual courses mentioning the terms (in the course’s title and/or course’s description) by the number of courses offered in the DCP. To prevent double counting, if a specific course mentioned a psychosocial related term in both the course title and course description it was counted only once toward the “number of courses with terminology mentioned”. Also, to prevent double counting of total courses listed in a DCP curriculum, if a course was titled “elective” with an implication that this course could be fulfilled by other listed courses, it was not counted toward the “total courses listed in DCP”.

To evaluate the CCE, Section 2 (Requirements for Doctor of Chiropractic Degree Educational Program), item H (Curriculum, Competencies and Outcomes Assessment) of their published Accreditation Standards document (2018) was queried using the same terminology as above, and the use of psychosocial terminology was tabulated.

To evaluate the NBCE, the test plans published for student review on their website (https://mynbce.org/prepare/) were queried using the same psychosocial terminology, and use of terminology was tabulated for parts I-IV. These test plans are organized by categories (e.g. general anatomy), subcategories (e.g. topographical anatomy), and topics of interest contained within subcategories. The NBCE provides the relative weight of each subcategory to the overall test category. For example, topographical anatomy has an 8% weighting of the general anatomy test. For this reason, we chose to emphasize the usage of psychosocial terms for test categories and subcategories for our data table and discussion, as this provides the most precise measure of inclusion. Any other mention of psychosocial terms further within test subcategories was collected to be described in the results only.

## Results

The term ‘public’ is the most commonly used term found across DCP curricula. The term ‘determinant’ was used in one (1) DCP curriculum. Occurrences of individual psychosocial related terms are represented in Supplementary Table 1. The frequency of psychosocial related terminology in DCP curricula is reported in Table 3. The number of courses with psychosocial related terminology in course titles ranged from 1 (Texas Chiropractic College) to 5 (Northwestern Health Sciences University). The number of courses with psychosocial related terminology used in the course descriptions ranged from 3 (multiple institutions) to 12 (National University of Health Sciences).

**Table 3:** Frequency of psychosocial related terminology in DCP curricula

Institution	# of course titles terminology used in	# of course descriptions terminology used in
Cleveland University Kansas City	4	3
D’Youville College	3	4
Keiser University	1	11
Life Chiropractic College West	3	8
Life University	3	11
Logan University	2	3
National University of Health Sciences (Illinois & Florida)	2	12
New York Chiropractic College	2	5
Northwestern Health Sciences University	5	11
Palmer College of Chiropractic – Iowa	2	3
Palmer College of Chiropractic – Florida	2	7
Palmer College of Chiropractic - West	3	6
Parker University	2	6
Sherman College of Chiropractic	2	11
Southern California University of Health Sciences	4	10
Texas Chiropractic College	1	3
Western States University	3	11
University of Bridgeport	4	4

The distribution and weighting of courses incorporating psychosocial related terminology amongst DCP curricula is described in Table 4. Most curricula had one (1) course with a description dedicated to psychology. Keiser University had none (0), and Northwestern Health Sciences University and Southern California University of Health Sciences each had two (2) dedicated psychology courses. Many institutions had one (1) or two (2) public health courses, whereas Texas Chiropractic College had none (0), and Cleveland University Kansas City, D’Youville College, National University of Health Sciences, and University of Bridgeport each had three (3). The remaining instances of terminology addressing psychosocial factors were contained in various clinical skills and special population (e.g. pediatrics, geriatrics) and other miscellaneous courses. With respect to the weighting of courses addressing psychosocial terminology as a percent of overall coursework, Palmer College of Chiropractic – Iowa had the lowest (3.40%) and Keiser University (14.86%) had the highest.

**Table 4:** Usage of psychosocial related terminology in DCP curricula

Institution	Total # of ***psych*** courses with terms used	Total # of ***public health*** courses with terms used	Total # of ***clinical skills/special pops/ other*** courses with terms used	# of courses with terms used	Total # of courses listed in DCP	% of courses containing searched terminology
Cleveland University Kansas City	1	3	0	4	105	3.80
D’Youville College	1	3	1	5	66	7.57
Keiser University	0	2	9	11	74	14.86
Life Chiropractic College West	1	2	5	8	129	6.20
Life University	1	2	8	11	182	6.04
Logan University	1	2	1	4	115	3.47
National University of Health Sciences (Illinois & Florida)	1	3	8	12	85	14.11
New York Chiropractic College	1	2	2	5	106	4.71
Northwestern Health Sciences University	2	1	8	11	112	9.82
Palmer College of Chiropractic - Iowa	1	1	1	3	88	3.40
Palmer College of Chiropractic – Florida	1	1	5	7	87	8.04
Palmer College of Chiropractic - West	1	2	4	7	95	7.36
Parker University	1	1	4	6	60	10.00
Sherman College of Chiropractic	1	1	9	11	138	7.97
Southern California University of Health Sciences	2	1	8	11	138	7.97
Texas Chiropractic College	1	0	2	3	61	4.91
Western States University	1	2	8	11	125	8.80
University of Bridgeport	1	3	2	6	89	6.74

The use of psychosocial related terminology within the CCE Accreditation Standards document, specific to their standard for DCP curricula, is shown in Table 5. A total of five (5) unique meta-competencies (out of 8 total) made mention of psychosocial related terminology. Psychosocial related terms were used a total of 17 times across these 5 meta-competencies.

**Table 5:** Usage of psychosocial related terminology in Council on Chiropractic Education Standards, Section 2, Item H (Curriculum, competencies, and outcomes assessment) [33]

Terminology	Total # of times term mentioned	Context of usage of psycho-social terminology
psychosocial	2	Standard H: Meta-Competency 1: Assessment & Diagnosis Standard H: Meta-Competency 3: Health Promotion And Disease Prevention
bio-psychosocial	1	Standard H: Meta-Competency 2: Management Plan
socio-economic	1	Standard H: Meta-Competency 4: Communication And Record Keeping
environmental	1	Standard H: Meta-Competency 3: Health Promotion And Disease Prevention
cultural	2	Standard H: Meta-Competency 4: Communication And Record Keeping
emotional	1	Standard H: Meta-Competency 5: Professional Ethics And Jurisprudence
public	5	Standard H: Meta-Competency 3: Health Promotion And Disease Prevention
community-based	1	Standard H: Meta-Competency 3: Health Promotion And Disease Prevention
behavior(s)	2	Standard H: Meta-Competency 2: Management Plan
behavioral	1	Standard H: Meta-Competency 3: Health Promotion And Disease Prevention

The frequency and weighting of psychosocial related terminology in NBCE parts I-IV test plans are described in Table 6. There were no (0) psychosocial related terms that served as primary test categories; four (4) terms were found within sub-categories. Of these four (4) items, one (1) was within the part I test plan, and the remaining three (3) were within part II. No psychosocial related terminology was found within parts III or IV. Two (2) additional items were found, as topics included within subcategories. These were “environmental and occupational toxicology” under the subcategory “toxicology”, and “emotional disorders and learning disabilities” under the subcategory “pediatrics”. Both subcategories are tested under “associated clinical sciences”, which is a component of the part II examination.

**Table 6:** Usage of psychosocial related terminology in test categories or subcategories for the National Board of Chiropractic Examiners parts I-IV test plans [34]

Psychosocial term	Part tested (I-IV)	Category belonging to	Topic weighting within category
*Environmental* and nutritional diseases	I	Pathology	5%
*Psychology*	II	Associated clinician sciences	11%
Occupational and *environmental* health	II	Chiropractic practice	9%
*Community* health and wellness	II	Chiropractic practice	11%

## Discussion

We present data on the prevalence of psychosocial related terminology within United States DCP curricula. Based on our findings, there is broad-spectrum use and emphasis on this topic amongst DCPs. We also describe the usage of psychosocial terminology by the CCE as it relates to their established curricular meta-competencies, and by the NBCE within their testing content.

There is a significant translational gap between knowledge of the contribution of psychosocial factors to SRDs and implementation in the training of all healthcare disciplines, including chiropractic. To point, a recent scoping review found substandard awareness amongst all health science students (e.g., chiropractic, physical therapy, medical) with respect to psychosocial factors and low back pain [8]. Without tangible changes at the educational level, this gap is unlikely to narrow. Because chiropractors largely treat SRDs, where trajectories of pain and disability are predominantly shaped by psychosocial, not biomedical factors, improvement of psychosocial education in DCP curricula is imperative [35, 36]. The chiropractic profession’s advocacy of a framework that recognizes the relationship between spinal health and the holistic state of the patient [37], (including the relationship between SRDs and socioeconomic status, lifestyle factors, and emotional health [3]), is only as viable as its commitment to addressing psychosocial factors as core drivers of health.

The allotment of course content to psychosocial topics varies widely within DCP curricula. This is important to address, as targeting education at the student level can modify clinical behavior [38]. Five out of 8 CCE meta-competencies address psychosocial factors. It is the authors’ interpretation that psychosocial factors appear to be largely addressed by the CCE within their curricular expectations, so the reason for the heterogeneity amongst DCP curricula studies is unclear. Perhaps, refining the objectives and outcomes of CCE meta-competencies, emphasizing definition, identification, and intervention associated with psychosocial factors could cultivate more homogeneity.

Based on our results, we are concerned by an emphasis on psychosocial topics within DCP curricula that may be insufficient to evoke meaningful change. Though it is challenging to determine what an acceptable level of content might be, even the programs with the highest number of psychosocial terms per total coursework didn’t clear 15%. In comparison, 2 United States DCPs have been found to use the term ‘subluxation’ in greater than 15% of their curricula, with 1 DCPs use measured at 19% [11]. Further comparison of our study findings shows that 9 United States DCPs use the term ‘subluxation’ in a greater percentage of their overall curriculum than psychosocial related terminology [11]. This level of inclusion of psychosocial terminology compared to ‘subluxation’ terminology may suggest to students a lack of importance of the BPS model compared to traditional dogma and potentially inhibits students from ascertaining and addressing the psychosocial factors of patients’ SRDs. Further, the term ‘determinant’ was used in only 1 DCP curriculum, which may risk leaving the causative link between psychosocial factors and health obscure for students.

The reasons for the lack of psychosocial wording within DCP curricula are complex and highly speculative, particularly given the selection bias inherent in using a course catalog to measure what is actually being taught. One explanation may be that some institutions have included a minimum acceptable level of content to pay “lip service” to the topic in order to satisfy CCE requirements, whereas others choose to devote more to the topic. This may be why psychosocial terminology is absent in NBCE parts III and IV, as representatives from DCPs provide suggestions for testing content. In the United States, parts III and IV tests are the final two standardized tests taken before seeking licensure and they are exclusively clinical and practical in nature. Thus, an implicit message to chiropractors just entering the SRD healthcare workforce could be that cursory knowledge of topics such as psychology, public and community health, and social determinants is sufficient for a practicing clinician and are merely “facts to be known” rather than factors illuminating “conditions to be challenged and changed.” [39] A radical change in both content and, especially, delivery, may be needed to shift away from students considering health as an isolated biomedical phenomenon and, instead, recognize the coalescence of biological, psychological and social determinants on health status, including those associated with human-made societal systems [39]. We argue that this change may be necessary to equip students to intervene with their patients on a true biopsychosocial level.

## Strengths and limitations

This is the first study that describes psychosocial components of DCP curricula in the United States. There are 3 noteworthy limitations. First, we are unable to determine the real time content being taught in DCP courses. Without access to course syllabi or actually observing course instruction, our assessment was limited to terminology used in DCP course titles and descriptions. It is possible that psychosocial content may be included in course instruction even though it is not mentioned in the official course title or description. Conversely, it is also possible that psychosocial content is not included in course instruction though it is mentioned in the official course title or description. This could lead to an under- or over-estimation of psychosocial related material presented in DCP curricula. Second, dedicated credit hours for each course were not included in all course catalogs and therefore were not included in our calculations, which may affect our measurements of psychosocial terminology use compared to the overall curriculum. Third, this study does not compare use of psychosocial related terminology to other educational programs and therefore our findings should be interpreted cautiously.

## Conclusions

Psychosocial related terminology is found in each DCP curriculum across the United States, however, the extent of DCPs’ use of terminology is variable. Psychosocial terminology is represented in a lower percentage of curricula than the term ‘subluxation’ at 9 DCPs. Psychosocial terminology appears in accreditation standards, though clinical and practical components of national pre-licensure examinations are completely devoid of it. These findings emphasize the need to understand the variability in psychosocial content in DCP curricula and how that affects not only student knowledge and skills but also awareness and use of the BPS approach amongst practicing clinicians. Results from this study can assist developers of DCP curricula in determining whether psychosocial aspects of health are being sufficiently integrated in their programs.

## Supplementary information

**Additional file 1: Supplementary Table 1.** Occurrences of individual psychosocial related terminology used in DCP curricula.

## Data Availability

All data collected are included and described in this manuscript.

## Abbreviations

BPS: Biopsychosocial
CCE: Council on Chiropractic Education
DCP: Doctor of Chiropractic Program
NBCE: National Board of Chiropractic Examiners
PDF: Portable Document Format
SRDs: Spine Related Disorders
