# Supplementary material for: The prevalence of psychosocial related terminology in chiropractic program courses, chiropractic accreditation standards, and chiropractic examining board testing content in the United States
Source: Chiropr Man Therap. 2020 Aug 21;28:43. doi: 10.1186/s12998-020-00332-7 (PMC7441694; doi:10.1186/s12998-020-00332-7)
Supplement: Supplementary file 1 — Additional file 1: Supplementary Table 1. Occurrences of individual psychosocial related terminology used in DCP curricula. [file 12998_2020_332_MOESM1_ESM.docx]

Supplementary Table 1. Occurrences of individual psychosocial related terminology used in DCP curricula.

| **Institution** | **Terminology** | **Frequency of use in DC curricula** |
| --- | --- | --- |
| Cleveland University Kansas City | psychology | 2 |
|  | psychological | 1 |
|  | mental | 1 |
|  | public | 5 |
|  | behavior | 1 |
| D’Youville College | psychology | 1 |
|  | bio-psycho-social-spiritual | 1 |
|  | environmental | 1 |
|  | mental | 1 |
|  | cultural | 1 |
|  | culture | 2 |
|  | public | 2 |
| Life Chiropractic College West | psychosocial | 1 |
|  | psychological | 2 |
|  | psychosomatic | 1 |
|  | psychosexual | 1 |
|  | social | 2 |
|  | dissociative | 1 |
|  | mental | 3 |
|  | environmental | 2 |
|  | cultural | 1 |
|  | public | 2 |
|  | community | 1 |
|  | behavior | 1 |
| Life University | psychology | 1 |
|  | psychoneuroimmunology | 1 |
|  | biosocial | 1 |
|  | psychosocial | 1 |
|  | environmental | 3 |
|  | mental | 2 |
|  | cultural | 1 |
|  | emotional | 1 |
|  | public | 3 |
|  | community | 1 |
|  | behavior(s) | 3 |
|  | behavioral | 3 |
|  | neurobehavioral | 1 |
| Keiser University | psychology | 1 |
|  | social | 2 |
|  | environmental | 1 |
|  | mental | 1 |
|  | cultural | 2 |
|  | culture | 1 |
|  | public | 5 |
| Logan University | psychology | 1 |
|  | social | 1 |
|  | environmental | 1 |
|  | public | 1 |
|  | community | 2 |
| National University of Health Sciences (Illinois & Florida) | psychopathology | 2 |
|  | psychology | 1 |
|  | psychological | 1 |
|  | sociological | 1 |
|  | society | 1 |
|  | psychosocial | 1 |
|  | sociodemographic | 1 |
|  | environmental | 2 |
|  | culturally-responsive | 1 |
|  | determinants | 6 |
|  | public | 4 |
|  | community | 1 |
|  | cognitive | 1 |
| New York Chiropractic College | psychology | 1 |
|  | psychological | 1 |
|  | psychosocial | 1 |
|  | neuropsychological | 1 |
|  | mental | 2 |
|  | environmental | 1 |
|  | emotional | 1 |
|  | public | 2 |
|  | community | 2 |
|  | behavior | 1 |
|  | behavioral | 1 |
| Northwestern Health Sciences University | social | 2 |
|  | society | 1 |
|  | psychological | 1 |
|  | psychology | 1 |
|  | environments | 1 |
|  | environmental | 2 |
|  | culture | 2 |
|  | cultural | 1 |
|  | behavioral | 1 |
|  | behaviors | 2 |
|  | public | 2 |
|  | community | 2 |
|  | mental | 3 |
| Palmer College of Chiropractic – Iowa | psychology | 1 |
|  | mental | 1 |
|  | environmental | 1 |
|  | cultural | 1 |
|  | public | 1 |
|  | community | 1 |
|  | communities | 1 |
| Palmer College of Chiropractic – Florida | psychology | 1 |
|  | psychological | 2 |
|  | psychosocial | 3 |
|  | public | 4 |
| Palmer College of Chiropractic – West | psychology | 2 |
|  | social | 2 |
|  | environmental | 2 |
|  | mental | 2 |
|  | public | 2 |
|  | behavior | 1 |
|  | behavioral | 1 |
| Parker University | psychology | 2 |
|  | psychological | 1 |
|  | psychosocial | 1 |
|  | social | 2 |
|  | environmental | 4 |
|  | mental | 1 |
|  | emotions | 1 |
|  | emotional | 1 |
|  | public | 2 |
|  | community | 1 |
|  | behaviors | 2 |
|  | behavioral | 1 |
| Sherman College of Chiropractic | psychosocial | 8 |
|  | psychological | 2 |
|  | psychology | 1 |
|  | non-mental | 1 |
|  | public | 2 |
|  | community | 1 |
|  | behaviors | 1 |
| Southern California University of Health Sciences | psycho | 1 |
|  | psychology | 3 |
|  | psychological | 6 |
|  | psychosocial | 1 |
|  | environmental | 2 |
|  | public | 2 |
|  | community | 1 |
|  | behavior(s) | 2 |
|  | behave | 1 |
| Texas Chiropractic College | psychology | 1 |
|  | psychological | 2 |
|  | psychotic | 1 |
|  | cultural | 1 |
| Western States University | psychology | 2 |
|  | psychological | 1 |
|  | psychopathology | 1 |
|  | biopsychosocial | 1 |
|  | psychoneurological | 1 |
|  | social | 1 |
|  | environmental | 1 |
|  | mental | 1 |
|  | emotion | 1 |
|  | emotional | 1 |
|  | public | 5 |
|  | behavioral | 1 |
|  | cognitive | 1 |
| University of Bridgeport | psychology | 1 |
|  | psychological | 1 |
|  | psychosocial | 2 |
|  | environmental | 1 |
|  | public | 4 |
|  | community | 2 |
|  | behavior | 1 |
